# T Helper Cell Subsets in the Pleural Fluid of Tuberculous Patients Differentiate Patients With Non-Tuberculous Pleural Effusions

**DOI:** 10.3389/fimmu.2021.780453

**Published:** 2021-12-02

**Authors:** Neda Dalil Roofchayee, Ian M. Adcock, Majid Marjani, Neda K. Dezfuli, Mohammad Varahram, Johan Garssen, Esmaeil Mortaz

**Affiliations:** ^1^ Department of Immunology, Faculty of Medicine, Shahid Beheshti University of Medical Sciences, Tehran, Iran; ^2^ Respiratory Section, National Heart and Lung Institute, Faculty of Medicine, Imperial College London, London, United Kingdom; ^3^ Clinical Tuberculosis and Epidemiology Research Center, National Research Institute of Tuberculosis and Lung Diseases, Shahid Beheshti University of Medical Sciences, Tehran, Iran; ^4^ Department of Immunology, School of Medicine, Dezful University of Medical Sciences, Dezful, Iran; ^5^ Mycobacteriology Research Center, National Research Institute of Tuberculosis and Lung Diseases (NRITLD), Shahid Beheshti University of Medical Sciences, Tehran, Iran; ^6^ Division of Pharmacology, Utrecht Institute Pharmaceutical Sciences, Faculty of Science, Utrecht University, Utrecht, Netherlands

**Keywords:** T helper, differentiation, frequency, tuberculous, pleural effusion

## Abstract

**Background:**

Tuberculous pleural effusion (TPE) is one of the most common forms of extrapulmonary tuberculosis (Tb). Patients with TPE or malignant pleural effusions (MPE) frequently have a similar lymphocytic pleural fluid profile. Since the etiology of PE in various diseases is different, identifying the cellular components may provide diagnostic clues for understanding the pathogenesis.

**Objective:**

We determined the frequency of T helper (Th) subtypes in the PEs for differentiation of Tb and non-Tb patients.

**Methods:**

Thirty patients with TPE, 30 patients with MPE, 14 patients with empyema (EMP), and 14 patients with parapneumonic effusion (PPE) were enrolled between December 2018 and December 2019. Five-milliliter fresh PE in tubes containing heparin as an anticoagulant was obtained from patients. The frequencies of CD4+IL-9+, CD4+IL-22+, CD+IL-17+, and regulatory T-cells CD4+CD25+ FOXP3+ (Treg) were determined by flow cytometry.

**Results:**

Treg cells have a lower frequency in TPE patients [4.2 (0.362–17.24)] compared with non-TPE patients [26.3 (3.349–76.93, p < 0.0001)]. The frequency of CD4+IL-9+ cells was significantly lower in TPE patients [3.67 (0.87–47.83)] compared with non-TPE groups [13.05 (1.67–61.45), p < 0.0001]. On the contrary, there was no significant difference in the frequency of CD4+IL-17+ and CD4+IL-22+ cells between TPE and non-TPE patients (p = 0.906 and p = 0.2188). Receiver-operator curve (ROC) analysis demonstrated that CD4+CD25+FOXP3+ T cells [optimal cutoff value = 13.6 (%), sensitivity 90%, specificity 75.86%] could be considered as predictor for TPE. However, adenosine deaminase [cutoff value 27.5 (IU/l), sensitivity 90%, specificity 96.5%] levels had an even greater predictive capacity.

**Conclusion:**

ADA, Treg cells, and CD4+IL-9+ cells may differentiate TPE from non-TPE patients. However, these results need validation in an independent large cohort.

## Introduction

Pleural effusions (PEs) are an accumulation of fluid between the pleural layers and are a clinical problem induced by several etiologies. These include local diseases of the pleura and diseases that result in increased pressure to the lung, organ dysfunction, systemic diseases, pulmonary infections, pleural tumor metastasis, and tuberculous pleurisy (1). Mycobacterium tuberculosis (*Mtb)* has two forms, pulmonary tuberculosis and extra-pulmonary tuberculosis (Tb). Tuberculous pleural effusion (TPE) is one of the most common etiologies of extra pulmonary Tb (2).

The differential diagnosis of TPE from other pleural effusions, especially malignant pleural effusion (MPE), is challenging clinically. TPE and MPE are both lymphocytic in origin (2, 3), and the gold standard for differentiating TPE from other pleural effusions with different etiologies is the isolation of *Mtb* from either pleural fluid or pleural biopsies (100% specificity) (4). Although culturing of sputum has a diagnostic value of 100% specificity, it is time consuming and delays the diagnosis. Manifestations of granuloma are also used to diagnose tuberculous pleurisy (~95%) provided other causes of granulomatosis are discounted (5).

TPE is a delayed hypersensitivity reaction related to activity of CD4+ T-cells, following the entry of TB antigens into the pleural space. An accumulation of lymphocytes, especially CD4+ T-cells, was seen in TPE (6–8). The T-helper (Th)1 cell‐mediated adaptive immune response to *Mtb* infection is very important but not enough to control the disease (9). T-cell subsets, such as Th17 cells (6, 10, 11), regulatory T-cells (Treg) (6), Th22 (10–12), and Th9 (13) cells, are implicated in the pathogenesis of TPE.

In the current study, we hypothesized that the frequency of T-cell subsets in PE will enable differentiation of TPE from non-TPE. Therefore, the frequency of T-cell subsets including CD4+IL-9+, CD4+IL-17+, CD4+IL-22+, and CD4+CD25+ FOXP3+ (Treg) cells in the PE was evaluated by flow cytometry. The frequency of Treg cells shows higher sensitivity, specificity, and predictive value to distinguish TPE from non-TPE patients.

## Materials and Methods

### Patient Selection

The study protocol was approved by the Institutional Review Board for human studies of the clinical center of the Masih Daneshvari Hospital, Tehran, Iran. Informed written consent was obtained from all subjects or their legal guardians. The study was carried out in accordance with the approved Ethics (ethic code: IR.SBMU.MSP.REC.1397.584). All experiments were performed in accordance with relevant guidelines and regulations. One hundred ninety consecutive patients with pleural effusions of unknown causes were enrolled between December 2018 and December 2019. After diagnosing the etiology of the PEs, 102 samples were excluded from the study as they failed to meet the diagnostic criteria (n = 30), provided transudate effusions (n = 45), or had exudates with miscellaneous etiology (n = 27). The number of patients with PEs with miscellaneous etiologies in each group was not suitable for statistical analysis. The remaining 88 patients with exudate PEs were classified into 4 diagnostic groups: TPE, MPE, empyema (EMP), and parapneumonic effusion (PPE).

Thirty HIV-negative patients, aged 18–84 years (13 female/17 male), with a positive *Mtb* test in biopsy specimens and pleural tissue granuloma were included in the TPE group. Thirty HIV-negative patients, aged 32–80 years (19 female/11 male), newly diagnosed with MPE based on histologically analysis (15 patients with adenocarcinoma, 10 patients with squamous cell carcinoma (SCC), and 5 patients with non-squamous cell carcinoma (NSCC) were included. Fourteen HIV-negative patients with EMP, aged 20–75 years (six female/eight male), and 14 HIV-negative patients with PPE, aged 35–83 years (five female/nine male), were included. The inclusion and exclusion criteria were as previously described (14).

PEs were classified into TPE, MPE, EMP, and PPE groups. TPEs fulfilled one or more of the following: (a) positive pleural fluid or pleural biopsy or sputum Ziehl–Neelsen stain or Lowenstein–Jensen culture and (b) caseous necrotic granulomas on pleural biopsy. MPEs were diagnosed by the discovery of malignant cells following pleural fluid cytology or pleural biopsy. No subjects with pleural mesothelioma or lymphoma were included in the study. Characterization of PEs from malignant patients was performed based on pathology of smears prepared by light microscopy. In addition, no samples from patients with a combination of MPE and TPE were included in the study.

EMPs were diagnosed by the presence of frank pus in their PE or positive bacterial or fungal culture of pleural fluid (except for *Mtb*). The diagnosis of PPEs was based on negative pleural fluid bacterial culture, pH < 7.2, and pleural fluid glucose < 600 (mg/dl).

### Sample Collection and Processing

The pleural fluid (5 ml) was collected by thoracocentesis into heparin-containing tubes within 24 h of hospitalization and immediately placed in ice as previously described (15). Tubes were centrifuged at 1,200×g for 5 min, and the pelleted cells were prepared for flow cytometry analysis.

Total and differential cell counts, protein, lactate dehydrogenase (LDH), adenosine deaminase (ADA), glucose, cytology, and bacterial examination were also evaluated.

### Flow Cytometry

To determine immunophenotyping of Th9, Th17, Th22, and Treg cells, surface staining of CD4 and CD25 markers was performed using mouse anti-human CD25-Alexa Fluor 488 (eBioscience, San Diego, CA, USA), CD4-PE (Immunostep, Salamanca, Spain), and CD4-FITC (BioLegend, San Diego, CA, USA) for 30 min at 4°C. Intracellular staining was followed by washing the cells and incubation with 1× fixation and permeabilization solution (BD Biosciences, San Diego, CA, USA) for 15 min at 4°C. The cells were subsequently washed with cold PBS, and intracellular staining was performed by mouse anti-human IL-17A-Alexa Fluor 660 (eBioscience, San Diego, CA, USA), mouse anti-human IL-9-PE (BioLegend, San Diego, CA, USA), mouse anti-human IL-22-PE (BioLegend, San Diego, CA, USA, Cat. No. 366703), and anti-human FOXP3-APC (eBioscience, San Diego, CA, USA) for 30 min at 4°C and kept in the dark. IgG1 Isotype-matched antibody controls were used for all staining. We used anti-human CD4-FITC for evaluation of CD4+IL-22+ and CD4+IL-9+ T cells, and anti-human CD4-PE for evaluation of Treg cells. Ten thousand events were evaluated with BD FACSCalibur (BD Biosciences) and analyzed using FlowJo Software version 10 (BD Company, USA).

### Statistical Analysis

Analysis was performed using SPSS version 16.0 (SPSS, Inc. Chicago, USA) and GraphPad Prism software (version 6; 07 GraphPad Software, Inc.). The non-parametric Mann–Whitney U test (median, 5%–95% percentile was used for the non-normally distributed variables and a t-test (mean ± SEM)) was used for normally distributed variables. For comparison between more than two groups, we used Kruskal–Wallis non-parametric ANOVA test with Dunn’s correction. Receiver operating characteristic (ROC) curve analysis was used to evaluate the capacity of ADA and the Th cell subset to differentiate TPE from non-TPE. The area under the ROC curve (AUC) was calculated, and 95% confidence intervals (CIs) were used to test the hypothesis that the AUC is 0.5. An optimum cutoff value was established by using the receiver operating Curve (ROC). p-values < 0.05 were considered as statistically significant.

## Results

**Figure 1:**
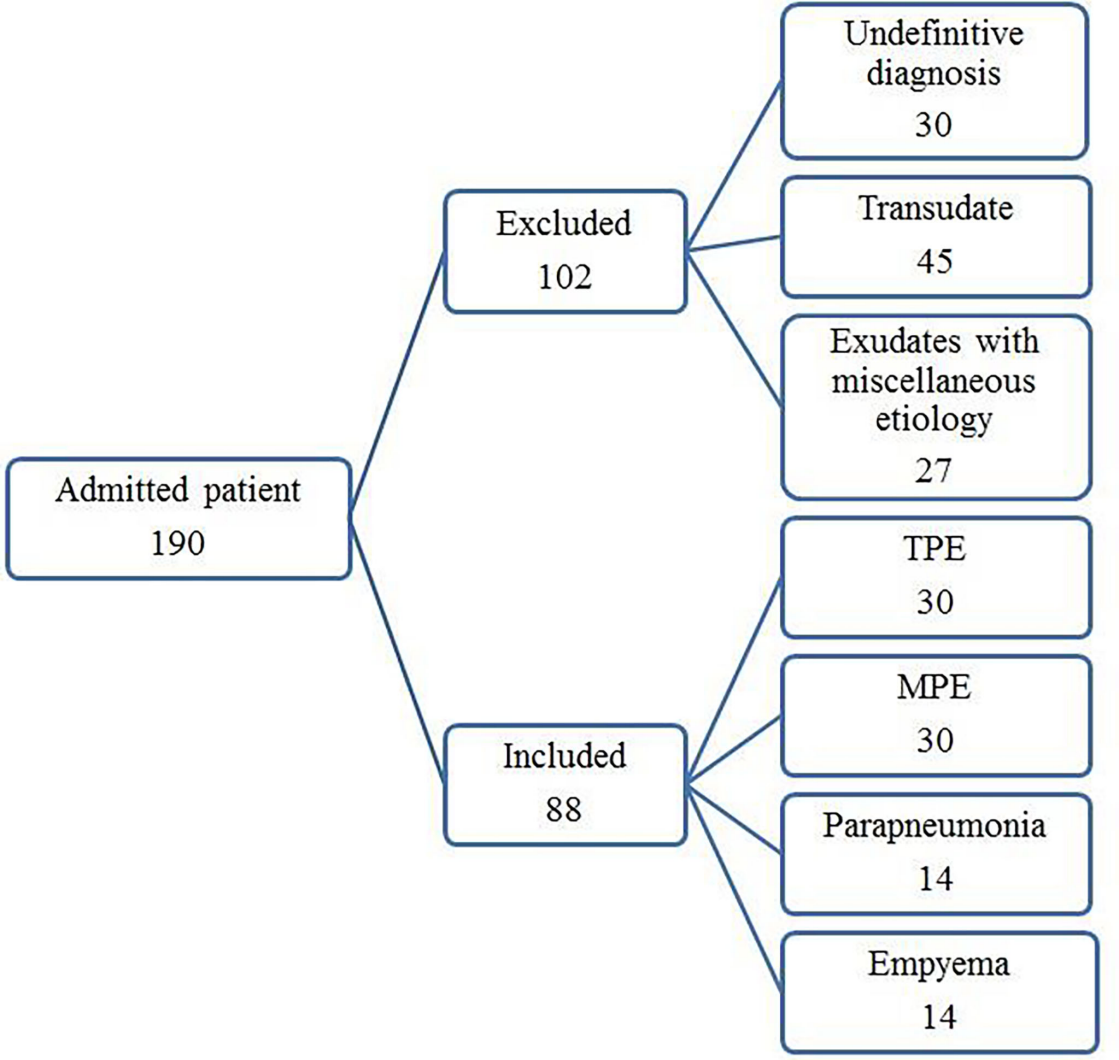
Flowchart.

The flowchart for patient recruitment shows the number of patients excluded from the study (102) and the number of subjects (88) included (**Figure 1**). There was no significant difference in age across the four subject study groups (TPE, MPE, EMP, and PPE), while the number of women in each group varied from 36% in the PPE group to 63% in the MPE group (**Table 1**). The LDH (p ≤ 0.0001) and protein concentrations (p = 0.0055) were significantly higher in TPE patients compared with non-TPE patients as a group (**Table 2**). However, although the LDH and protein levels were lower in the MPE (p < 0.0001 and p = 0.0105, respectively) and PPE (p < 0.0001 and p = 0.0248) groups compared to the TPE subjects, the levels were similar in the EMP subjects (p = 0.4228 and p = 0.8928, respectively) (**Table 2**). ADA levels were higher in the TPE group compared with MPE (p < 0.0001), EMP (p < 0.0001), PPE (p < 0.0001), and non-TPE groups (p < 0.0001) (**Table 2**).

**Table 1:** Demographic data of the study population.

	Patients	TPE	MPE	EMP	PPE	p value^†^
**N**	88	30	30	14	14	–
**Age, year**	56.6 ± 1.9	52.3 ± 3.9	59.5 ± 2.8	56.0 ± 4.2	59.9 ± 3.2	0.3676
**Sex (F/M), n**	43/45	13/17	19/11	6/8	5/9	–

Values are presented as mean ± standard error of the mean (SEM). TPE, tuberculous pleural effusion; MPE, malignant pleural effusion; EMP, empyema; PPE, parapneumonic pleural effusion.

^†^Comparisons of data between TPE, MPE, EMP, and PPE effusions were performed using one-way analysis of variance.

**Table 2:** Biochemical and cytological characteristics of pleural effusions.

	TPE	Non-TPE	p value*				
**LDH (IU/L)**	631 ± 19.7*	456.4 ± 17.5	≤0.0001				
**Protein (g/L)**	39.2 ± 1.78	33.40 ± 1.14	0.0055				
**ADA (IU/L)**	42.73 ± 1.71	18.9 ± 0.07	≤0.0001				
	**TPE**	**MPE**	**p value (TPE *vs*. MPE)**	**EMP**	**p value (TPE *vs*. EMP)**	**PPE**	**p value (TPE *vs*. PPE)**
**LDH (IU/L)**	631.9 ± 19.7	382.8 ± 10.6	<0.0001	657.4 ± 18.2	0.4228	413.2 ± 24.4	<0.0001
**Protein (g/L)**	39.2 ± 1.8	33.0 ± 1.5	0.0105	35.9 ± 2.3	0.8928	31.8 ± 2.6	0.0248
**ADA IU/L**	42.73 ± 1.71	14.9 ± 0.61	≤0.0001	25 ± 1.11	≤0.0001	21.21 ± 0.32	≤0.0001
**Differential cell counts, %**							
**Lymphocytes cells**	72.8 ± 1.07	53.5 ± 0.7	≤0.0001	14.4 ± 0.8	≤0.0001	19.3 ± 0.9	≤0.0001
**Neutrophils cells**	8 ± 0.4	5.9 ± 0.3	≤0.0001	78.4 ± 0.9	≤0.0001	53.7 ± 1.1	≤0.0001
**Macrophage cells**	12.5 ± 0.4	31.2 ± 0.4	≤0.0001	5.7 ± 0.5	≤0.0001	8.6 ± 0.5	≤0.0001
**Mesothelial cells**	3.1 ± 0.2	6.6 ± 0.3	≤0.0001	5.7 ± 0.3	≤0.0001	1.6 ± 0.3	0.0001
**Malignant cells**	–	3.5 ± 0.3	–	–	–	–	–

Values are presented as mean ± standard error of the mean (SEM).

ADA, adenosine deaminase; LDH, lactate dehydrogenase; TPE, tuberculous pleural effusion; MPE, malignant pleural effusion; EMP, empyema; PPE, parapneumonic pleural effusion.

*Values are shown as mean ± SEM. Comparisons of data between TPE and non-TPE groups were performed using Student’s t test.

The lymphocyte levels in TPE patients were significantly higher than in EMP (p < 0.0001), PPE (p < 0.0001), and MPE patients (p < 0.0001). In addition, a neutrophil and macrophage percentage varies between groups (**Table 2**). Neutrophil numbers in TPE patients were significantly higher than in MPE subjects (p < 0.0001) but significantly lower than in EMP (p < 0.0001) and PPE (p < 0.0001) subjects. Conversely, macrophage numbers were significantly higher in MPE (p < 0.0001) but significantly lower in EMP (p < 0.0001) and PPE (p < 0.0001) patients compared with TPE patients (**Table 2**).

### The Frequency of T Helper Subsets in TPEs and Non-TPEs

**Figure 2:**
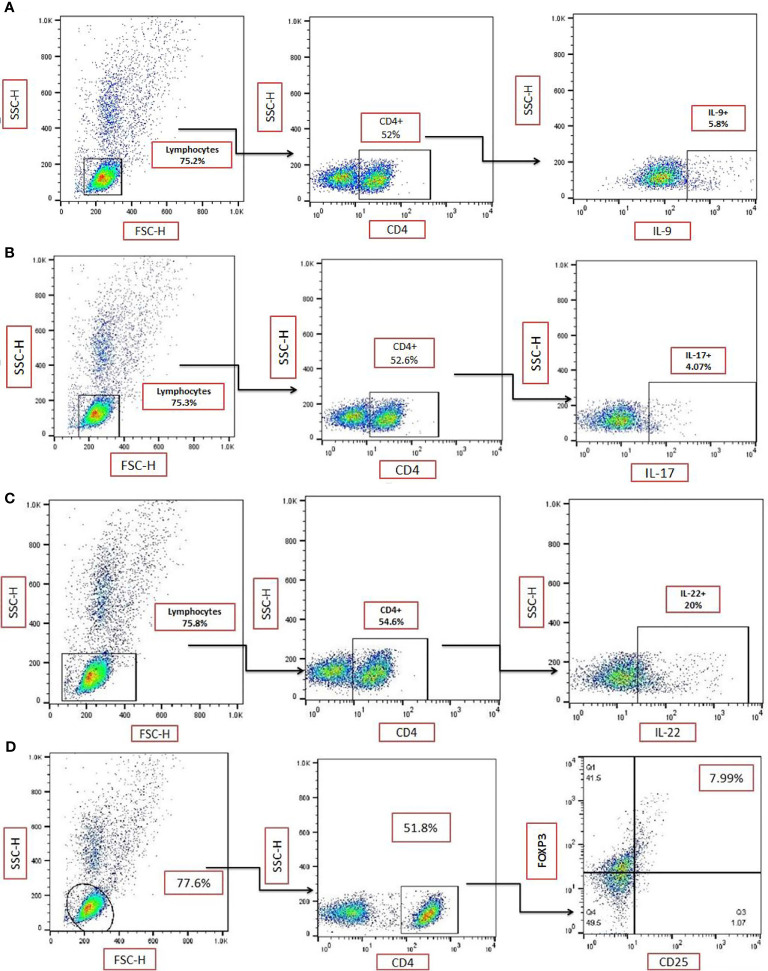
Gating strategy for identification of T-cell subsets. Representative flow cytometric dot plots showing expression of **(A)** IL-9 on CD4+ T-cells in a sample from a patient with a tuberculous pleural effusion (TPE). **(B)** Representative flow cytometric dot plots showing the expression of IL-17 on CD4+ T cells in a single TPE sample. Data from **(A, B)** are from duplicate samples from the same donor. **(C)** Representative flow cytometric dot plots showing the expression of IL-22 on CD4+ T cells in a TPE sample. **(D)** Representative flow cytometric dot plots showing expression of CD25+FOXP3+ on CD4+ T cells in a TPE subject. TPE, tuberculous pleural effusion.

**Figure 3:**
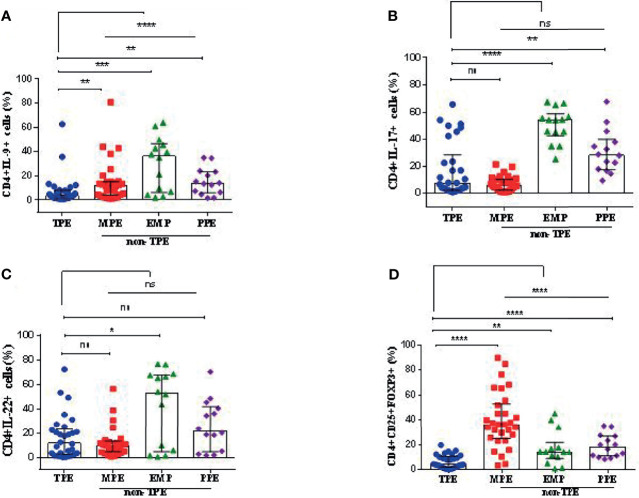
Graphical analysis of T-cell subset frequencies in pleural effusions. **(A)** The frequency of CD4+IL-9+ cells in TPE (n = 30), MPE (n = 30), EMP (n = 14), and PPE (n = 14). **(B)** The frequency of CD4+IL-17+ cells in TPE (n = 30), MPE (n = 30), EMP (n = 14), and PPE (n = 14). **(C)** The frequency of CD4+IL-22+ cells in TPE (n = 30), MPE (n = 30), EMP (n = 14), and PPE (n = 14). **(D)** The frequency of CD4+CD25+FOXP3+ cells (Treg cells) in TPE (n = 30), MPE (n = 30), EMP (n = 14), and PPE (n = 14). TPE, tuberculous pleural effusion; MPE, malignant pleural effusion; PPE, parapneumonic pleural effusion; EMP, empyema. All values are presented as the median and 5%–95% percentile and comparisons made between TPE, and the other groups were performed using the Mann–Whitney U test. *p < 0.05, **p < 0.01, ***p < 0.001, ****p < 0.0001. ns, non significant.

The strategy for gating for CD4+IL-9+ (**Figure 2A**), CD4+IL-17+ (**Figure 2B**), CD4+IL-22+ (**Figure 2C**), and CD4+CD25+FOXP3+ (**Figure 2D**) in TPE samples was shown. The frequencies of CD4+ IL-9+, CD4+IL-17+, CD4+IL-22+, and CD4+CD25+FOXP3+ T cells in the TPE and non-TPE (MPE, EMP, PPE) groups were analyzed in the PE (**Table 3** and **Figure 3**).

**Table 3:** The frequency of CD4+IL-9+, CD4+IL-17+, CD4+ IL-22+, and CD4+CD25+FOXP3+ cells in the pleural effusions.

	TPE	MPE	p value	EMP	p value	PPE	p value	p value* TPE vs. MPE, PPE, PPE	Non-TPE	P value
**CD4+IL-9+ (%)**	3.67 (0.87–47.83)	11.5 (1.386–60.54)	0.002	36.15 (2.12–64.2)	0.0005	13.75 (1.69–34.8)	0.0029	0.0003	13.05 (1.67–61.45)	<0.0001
**CD4+IL-17+ (%)**	7.15(1.118– 9.43)	5.6 (1.046–20.27)	0.2343	54.25 (25.2–67.5)	<0.0001	28.2 (9.31–67.5)	0.0054	<0.0001	15.05 (1.195 -66.55)	0.0906
**CD4+IL-22+ (%)**	0.2 (0.185–61.75)	9.2 (0.871–46.62)	0.6517	52.9 (0.34–76.5)	0.0241	21.85 (1.57–70.2)	0.1148	0.03	11.8 (1.119–70.5)	0.2188
**CD4+CD25+FOXP3+ (%)**	4.2 (0.362-17.24)	36.15(4.088-87.21)	<0.0001	14.2 (0.86-45.30)	0.0023	40.3 (8.42–35)	<0.0001	<0.0001	26.3 (3.349–76.93)	<0.0001

All values are presented as the median and 5%–95% percentile and comparisons made between TPE and the other individual groups, and the combined non-TPE groups were performed using the Mann–Whitney U test.

*A Kruskal–Wallis non-parametric test with Dunn’s correction made while the comparison between TPE and the other groups (MPE, PPE, and MPE).

The median of CD4+IL-9+ frequency in the TPE group was 3.67% (5%–95% percentile, 0.87%–47.83%) which was significantly lower compared with the non-TPE group (13.05%, 5%–95% percentile; 1.68–61.45, p < 0.0001) (**Table 3** and **Figure 3A**). The median CD4+IL-9+ cell percentages in MPE, PPE, and EMP patients are shown in **Table 3** and **Figure 3A**.

The frequency of CD4+IL-17+ cells in TPE patients [7.15% (5%–95% percentile, 1.12%–59.43%)] did not differ from non-TPE patients [15.05%, (5%–95% percentile; 1.20%–66.55%), p = 0.0906] (**Table 3** and **Figure 3B**). The median of CD4+IL-17+ cell percentage in MPE, PPE, and EMP patients is shown in **Table 3** and **Figure 3B**.

The frequency of CD4+IL-22+ cells in the TPE group [11.7% (5%–95% percentile; 0.185%–61.75%)] was similar to that in the non-TPE group [11.8% (5%–95% percentile; 1.119%–70.5%), p = 0.2188] (**Table 3** and **Figure 3C**). In addition, the median of the CD4+IL-17+ cell percentage in MPE, PPE, and EMP patients is shown in **Table 3** and **Figure 3C**.

The percentage of CD4+CD25+FOXP3+ T cells (Treg) in the TPE patients [4.2% (5%–95% percentile, 0.362% to 17.24%)] was significantly lower than in the non-TPE group [26.3%, 5%–95% percentile; 3.349%–76.93%), p < 0.0001] (**Table 3** and **Figure 3D**). The frequencies of CD4+CD25+FOXP3+ T cells in the TPE, PPE, MPE, and EMP subjects are shown in **Table 3** and **Figure 3D**.

### ROC AUC Analysis to Determine Predictive Values of T-Cell Subsets in TPE

**Figure 4:**
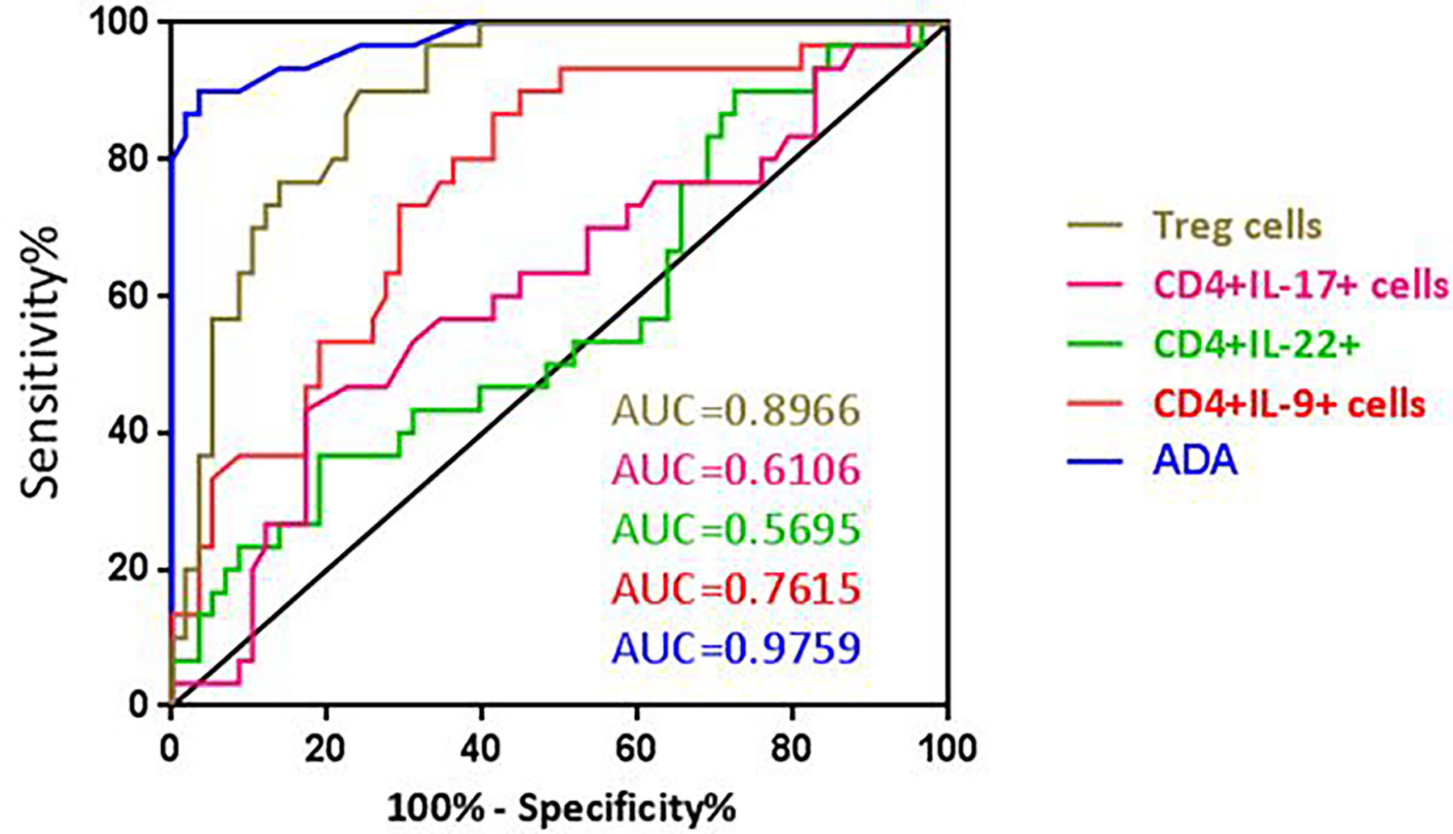
ROC curve of ADA, CD4+IL-9+, CD4+IL-17+, CD4+IL-22+, and Treg cells for differential diagnosis of TPE (n = 30) versus non-TPE (n = 58).

We determined the optimal CD4+IL-9+ cutoff value of 12.35% in the pleural fluid by the ROC curve. With this cutoff value, a sensitivity of 90% (95% CI: 73.47% to 97.89%) and a specificity of 55.1% (95% CI: 41.54% to 68.26%), together with a positive likelihood ratio (PLR) = 2, a negative likelihood ratio (NLR) = 0.18, a positive predictive value (PPV) = 50.94, and a negative predictive value (NPV) = 91.42 for TPE diagnosis, were obtained compared with non-TPEs. Thus, the diagnostic accuracy of CD4+IL-9+ frequency in pleural effusion was 67.04% (59/88) (**Table 4** and **Figure 4**).

**Table 4:** The diagnostic accuracy of CD4+IL-9+, CD4+IL-17+, CD4+IL-22+, CD4+CD25+FOXP3+ T cells, and ADA in the differentiation of tuberculous from non-tuberculous effusions (malignant, empyema, and parapneumonic effusions).

Variables	Cutoff value	AUC (95% CIl)	p value	Sensitivity (%)	Specificity (%)	Positive likelihood ratio	Negative likelihood ratio	Positive predictive value	Negative predictive value	Diagnostic accuracy (%)
**TPE and non-TPE**										
**CD4+IL-9+ (%)**	<12.35	0.7615	<0.0001	90	55.17	2	0.18	50.94	91.42	67.04
**CD4+IL-17+ (%)**	<8.55	0.6106	0.0902	56.67	65.52	1.64	0.66	31.48	61.76	43.18
**CD4+IL-22+ (%)**	>35.6	0.5694	0.2868	90	27.59	1.24	0.36	39.13	84.21	48.86
**CD4+CD25+FOXP3+ (%)**	<13.6	0.8644	<0.0001	90	75.86	3.729	0.131	65.85	93.61	80.68
**ADA**	>27.5 IU/l	0.9759	≤0.0001	90	96.5	26.1	0.1	93.1	94.9	94.3

AUC, area under the curve; CI, confidence Interval.

The optimal CD4+IL-9+ and CD4+IL-22+ cutoff values, sensitivity, specificity, PLR, NLR, PPV, NPV, and diagnostic accuracy for diagnosing TPE are also shown in **Table 4** and **Figure 4**.

The optimal CD4+CD25+FOXP3+ (Treg) cell cutoff value was calculated as 13.6% for PE using the ROC curve. This gave a sensitivity of 90% (95% CI: 73.47% to 97.89%), a specificity of 75.86% (95% CI: 62.83% to 86.13%), PLR = 3.729, NLR = 0.131, PPV = 65.85, and NPV = 93.61 which were obtained for differentiating TPE from non-TPEs. The diagnostic accuracy of these cells for TPE diagnosis was 80.68% (71/88) (**Table 4** and **Figure 4**).

### ADA Levels Can Discriminate Between TPE and Non-TPE

The AUC for ADA to differentiate between TPE and non-TPE was 0.975 (95% confidence interval, 0.9471 to 1.005; p ≤ 0.0001). With a cutoff value of 27.5 (IU/l), we obtained a sensitivity of 90% and a specificity of 96.5%, together with a PLR = 26.1, a NLR = 0.1, a PPV = 93.1, a NPV = 94.9, and a diagnostic accuracy of 94.3% (**Table 4** and **Figure 4**).

## Discussion

The current study demonstrated that the frequencies of CD4+IL-9+ and Treg cells were significantly lower in the pleural fluid of TPE compared with non-TPE participants. The specific AUC cutoff values for ADA levels and Treg cells were identified, and these had good sensitivity and specificity for TPE against non-TPE subjects. The ADA diagnostic value was higher than that for Treg cells. In contrast, the frequencies of CD4+IL-17+ and CD4+IL-22+ T-cells were similar between TPE and non-TPE subjects. We also confirmed that ADA levels were significantly elevated in TPE compared with non-TPE subjects and that LDH levels were elevated in TPE subjects compared to other non-TPE groups except for those with EMP. TPE samples also contained elevated percentages of lymphocytes compared with samples from non-TPE subjects and correspondingly low neutrophil percentages compared with non-TPE subjects.

Various analytes within PE are currently being studied as potential biomarkers of disease etiology including ADA, LDH, CRP, and IFN-γ. For example, 40 IU/ml ADA in PE has a sensitivity (81%–100%) and specificity (83%–100%) for TPE (15). Reducing the ADA cutoff value to >35 U/ml results in a lower sensitivity (93%) and specificity (90%) for diagnosing TPE (16). In a systematic review, Aggarwal and colleagues examined 174 publications with 27,009 patients. Importantly, all studies had a high risk of bias but suggested good sensitivity (0.92), specificity (0.9), and diagnostic odds ratio (97.42). Many studies (65) used an ADA threshold of 40 ± 4 (IU/l) which gave a good sensitivity (0.93) and specificity (0.90) while four studies using an ADA threshold of >65 (IU/L) gave a sensitivity and specificity of 0.86 and 0.94, respectively. An earlier meta-analysis indicated that the summary measure derived from ROC curves was 92.2% for both sensitivity and specificity (17). In addition, the expression of CCL27 and of CD4^+^CCR10^+^ T cells within PF may also help in diagnosing TPE in patients with moderate elevation of PF ADA levels (18).

In the current study, ADA, LDH, and pleural fluid protein levels were higher than in TPE patients than in non-TPE patients, and these together with a higher lymphocyte and lower neutrophil frequency may aid the differentiation of TPE from non-TPE patients.

Treg cells from both murine and humans are CD4+ and express high levels of the IL-2Rα chain (CD25) and the transcription factor Foxp3 and low expression of IL-7R (CD127) (19, 20). The importance of Treg cells in tuberculosis has been demonstrated (20, 21), although their specific role during tuberculosis is not well understood but may involve restricting the “strong” Th1 responses induced by microbial antigens and to prevent excessive inflammation and tissue damage (22) and enhanced activation of CD4+ CD25+ Treg cells may negatively modulate anti-TB immune responses (23). Previous data have indicated similar levels of Treg cells in the PE of TPE patients compared to MPE subjects (22). The wide spread of Treg cells seen in our study suggests that subtypes of patients with MPE may have very high levels of Treg cells or that ongoing therapies may affect the levels of Treg cells in these patients. Further studies are needed to address this issue.

Ye and colleagues have shown higher frequencies of both Th17 and CD39+ Treg cells in TPE (6). Th17 cell numbers were correlated negatively with Tregs in TPE but not in blood. When naïve CD4+ T cells were cultured with CD39+ Tregs, Th17 cell numbers decreased as CD39+ Treg numbers increased. Overall, the data suggest that there is a Th17/Treg imbalance in TPE and that pleural CD39+ Tregs inhibit the generation and differentiation of Th17 cells *via* a latency-associated peptide-dependent mechanism (6). Caramori and colleagues reported that Th cells (Th1/Th2/Th17/Treg cells) in biopsies of layers of pleura obtained from TPE patients show higher CD3+, CD4+, CCR4+, and Th17 cells (24). They described lower frequencies of mast cells and GATA-3-positive T cells in the parietal pleura layer and indicated that this may account for the differentiation of TPE from others from nonspecific pleurisy (24).

There was a higher frequency of CD4+CD25+ T cells in patients with lung cancer and PE than in subjects with lung cancer without PE (25). Our current study shows that Treg cells were significantly lower in TPE patients than non-TPE patients. Using ROC analysis, a CD4+CD25+FOXP3+ T cell cutoff value <13.6% had a diagnostic accuracy of 81%. The increased percentage of CD+4CD25+ T cells in TPE might be due to active recruitment or local differentiation. This may also reflect the lower levels of CCL22 in PE since this chemokine plays an important role in recruiting Treg cells (23).

Furthermore, we showed that the frequency of Th9 cells was lower in TPE than in non-TPE patients, confirming previous reports (13). It is reported that these cells develop following TGFβ exposure of CD4+ precursor cells (26). Ye and colleagues compared the frequency of these cells in the pleural effusion and blood of patients with tuberculous pleurisy and found a higher frequency of Th9 cells in TPE than blood (13). An *in vitro* study showed that increased expression of IL-9 may contribute to the development of tuberculosis. Since PMCs are an important component of the pleural environment, they may interact with other cell types, including Th9 cells, to instigate local cell-mediated immunity against M. tuberculosis. The mesothelium is a slowly renewing tissue that can be stimulated by a variety of agents as well as by direct physical damage to increase its turnover rate and enhance fibrosis (27, 28). The current study suggests that CD4+IL-9+ T cells may predict TPE compared to non-TPE subjects with 90% sensitivity and a specificity of 55.17%. Our data suggest that compared to patients without TPE, patients with tuberculous pleurisy have a two-fold higher chance of being CD4+IL-9+ T cell positive. Similarly, if a patient has a negative result of CD4+IL-9+ T cells, he would have a 0.18% chance of being a tuberculous pleurisy patient. However, its diagnostic value (67.04%) was much less than that of CD4+CD25+FOXP3+ T cells and ADA.

Ye and colleagues reported that IL-22-producing CD4+ cells were elevated in MPE patients possibly due to the local production of pleural cytokines and chemokines (15). In turn, Th22 cells exert important immunomodulatory effects on cancer cells in the human pleural malignant environment (29). In our study, the frequency of CD4+IL-22+ in TPE patients did not differ from that in MPE and PPE subjects; however, the frequency of these cells was significantly lower in TPE patients compared with EMP patients. Th22 cells are significantly higher in TPE than in blood (12), which suggests that local pleural cytokines and pleural mesothelial cells and their mediators are responsible for these effects (12). For example, pleural mesothelial cells are able to act as antigen-presenting cells enabling the stimulation of CD4+ cell proliferation and Th22 cell differentiation (12). In addition, we have previously shown that the levels of ADA, CCL1, CXCL-8, IL-6, IL-27, and IP-10 in pleural fluid were significantly higher in TPE compared to non-TPE patients (14). The actions of these mediators may account for differential frequencies of CD4+ IL-9+ and Treg cells between TPE and non-TPE patients.

Although there are several strengths to our study, we recognize that there are some limitations. These include the low number of participants, the limited range of T cell subsets and mediators investigated, and the lack of a validation cohort. In mitigation, the study is as large as many other studies attempting to differentiate between TPE and non-TPE, and we were able to clearly differentiate between TPE and non-TPE subjects including subsets of non-TPE patients using a combination of biochemical, cytological, and FACs analyses. In addition, methodologically it would have been optimal to have used an anti-CD3 antibody rather than an anti-CD4 antibody to identify the T helper lymphocyte population. However, this was not available to us during the pandemic. Future studies are required in a larger multicenter cohort to validate these results.

In conclusion, current data suggest that the determination of ADA, CD4+IL-9+, and Treg cells in pleural effusion may be an important diagnosis marker for TPE. However, it needs to be extended extensively by multicenter studies for possible usage as biomarker for the development of a rapid and non-invasive diagnostic test for differentiation TPE from non-TPE.

## Data Availability Statement

The original contributions presented in the study are included in the article/supplementary material. Further inquiries can be directed to the corresponding authors.

## Ethics Statement

The studies involving human participants were reviewed and approved by the Institutional Review Board for human studies of clinic center from Masih Daneshvari Hospital, Tehran, Iran. The patients/participants provided their written informed consent to participate in this study.

## Author Contributions

EM and MM designed the study and drafted the manuscript. ND and NK did the flow cytometry and analyzed the data. MM and MV recruited the patients and collected their clinical data. IA and JG edited the manuscript and revised the English skills and grammar. All authors contributed to the article and approved the submitted version.

## Funding

IA is supported by the EPSRC (EP/T003189/1 and EP/V052462/1), the UK MRC (MR/T010371/1 and MR/M016579/1) and the Wellcome Trust (208340/Z/17/Z). IA are supported by the Imperial College Jameel Trust for work on COVID-19.

## Conflict of Interest

The authors declare that the research was conducted in the absence of any commercial or financial relationships that could be construed as a potential conflict of interest.

## Publisher’s Note

All claims expressed in this article are solely those of the authors and do not necessarily represent those of their affiliated organizations, or those of the publisher, the editors and the reviewers. Any product that may be evaluated in this article, or claim that may be made by its manufacturer, is not guaranteed or endorsed by the publisher.
